# Nut and Bolt Microfluidics with Helical Minichannel for Counting CD4+ T-Cells

**DOI:** 10.3390/bioengineering6010024

**Published:** 2019-03-15

**Authors:** Jung Kyung Kim, Mohiuddin Khan Shourav, Myoung-Ock Cho, Yein Lee

**Affiliations:** 1 School of Mechanical Engineering, Kookmin University, 77 Jeongneung-ro, Seongbuk-gu, Seoul 02707, Korea; fallslover@naver.com; 2 Department of Mechanical Engineering, Graduate School, Kookmin University, 77 Jeongneung-ro, Seongbuk-gu, Seoul 02707, Korea; khan@kookmin.ac.kr (M.K.S.); yeinlee20@gmail.com (Y.L.)

**Keywords:** nut and bolt microfluidics, helical minichannel, cell counter, sample cartridge, HIV/AIDS, CD4+ T-cells, fluorescence imaging

## Abstract

In this study, we developed the prototype of an optical imaging-based point-of-care (POC) device for monitoring human immunodeficiency virus (HIV)/acquired immunodeficiency syndrome (AIDS) progression that can detect CD4+ T-lymphocytes in human blood. The proposed portable cell-counting system, Helios CD4 Analyzer (Helios), can acquire sample images and analyze the cells automatically using a simple fluorescence imaging module and sample cartridge with a three-dimensional (3D) helical minichannel. The helical minichannel formed on the cylindrical surface enables the sample cartridge to hold a cell suspension present in a fixed sample volume for absolute counting of the cells. With a given total channel length, the helical minichannel-based sample cartridge is smaller than the conventional sample cartridge with a planar microchannel. The implemented nut and bolt mechanism allows the scanning of a relatively large volume of the sample along the helical minichannel by just rotating the cylindrical chamber coupled with a single DC motor rather than using a two-axis motorized translation stage, which considerably simplifies the associated electromechanical parts. It has distinct advantages over the existing devices because of its small size and simple scanning mechanism. We optimized various imaging parameters to enhance the fluorescence detection efficiency of the prototype. Performance evaluations using human blood samples demonstrated good agreement for low CD4 count between the Helios and the PIMA^TM^, one of the most widely used POC CD4+ analyzers.

## 1. Introduction

The 2014 Joint United Nations Program on HIV/AIDS (UNAIDS) report states that approximately 37 million people worldwide are infected with the human immunodeficiency virus (HIV) that can lead to acquired immunodeficiency syndrome (AIDS). HIV destroys CD4+ T-lymphocytes (CD4 cells) in blood, which are T helper cells expressing CD4 (cluster of differentiation 4) molecules on their surface and protecting the human immune system. As a result, the number of CD4 cells reduces gradually in patients with HIV/AIDS. A normal person has more than 500 CD4 cells in 1 μL of blood, however; HIV-infected patient has lesser number of CD4 cells. The World Health Organization (WHO) recommended that adults with less than 350 CD4 cells should be treated with antiretroviral therapy (ART). Majority patients with HIV/AIDS are concentrated in developing regions such as Africa and Southeast Asia. AIDS-related mortality has been declining owing to the introduction of the ART [1,2]. However, the gold standard method for monitoring CD4 cell count, i.e., flow cytometry, is complicated and expensive to use, especially in the areas where resources are limited [3]. Therefore, new methods for counting CD4 cells based on image cytometric analysis have emerged. Many types of microfluidic point-of-care (POC) devices have been developed for monitoring HIV/AIDS by counting CD4 cells. POC devices are defined as analytical or diagnostic instruments that can perform tests at the site of interest quickly as well as easily and provide results immediately after testing [4]. However, researchers continue to develop more efficient low-cost devices that can enhance patient comfort by reducing the number of visits to clinics and using gentler blood sampling owing to a reduced blood volume requirement for monitoring HIV/AIDS in resource-poor settings [5]. The PIMA (Pima^TM^ CD4 Analyzer, Alere, Waltham, MA, USA), one of the commercially available portable CD4 analyzers, has been widely used around the world; it is also considered to be an alternative to the conventional flow cytometry due to its compact size and convenient use. However, it is expensive to perform the test each time and has a low throughput of only 15 tests per day. The cost per test using the PIMA is estimated to be 18–24 USD approximately considering various individual pieces of information such as material cost and labor cost [6]. Another bench-top fluorescence cell counter, ADAMII (NanoEntek, Seoul, Korea), has similar size and performance like the PIMA, but it is costly to repeatedly perform the test owing to the cartridge kit and has a low throughput [7]. The cost can be slightly reduced using Dynal T4 Quant Kit (Thermo Fisher Scientific, Waltham, MA, USA) but it is unsatisfactory, thereby justifying the continued development for more effective, fast, and reliable devices. It is believed that the cost per test can be further reduced by decreasing material cost using a simple cartridge, and the throughput can be increased further by minimizing the time required for acquisition and analysis of the cell images. We aim to contribute to these efforts by focusing on improving the situation in developing countries and ultimately promoting human health by developing a portable CD4 cell analyzer for monitoring the effectiveness of the ART for patients with HIV/AIDS.

A sample cartridge that is capable of holding a certain volume of the sample is required to measure particle concentration by analyzing particle images obtained using a camera [8]. Particle concentration (number/μL) is determined as the total number of particles contained in a sample divided by the sample volume. Considering the depth of focus of a typical optical system, the height of the channel of the sample detection unit is restricted to 100 μm or less. Therefore, an area of 200–300 mm^2^ or more is required to accommodate 20–30 μL of the sample. To increase the sample volume to obtain the statistical significance of the measured values, the samples included in a larger area should be photographed [9]. Therefore, a two-axis translation stage for scanning the detection channel should be equipped [10]. However, two motors with the translation stage are too bulky to be mounted on a mobile device like a smartphone, which is a next-generation handheld analyzer. Furthermore, interface applications and battery capacity for controlling and driving two motors are not yet fully supported. Hence, a new type of sample cartridge is required to develop a POC system that uses mobile devices as analyzers.

We have recently devised a three-dimensional (3D) helical minichannel-based cylindrical sample cartridge to replace the conventional sample chamber containing a planar microchannel [11]. Our sample cartridge has simplified the design of the mechanical unit of the analyzer using a single DC motor instead of a two-axis translation stage, which has enabled us to analyze a larger sample volume by capturing particle images along a helical minichannel based on a nut and bolt mechanism. In the present study, we aim to optimize various imaging parameters for the development of a prototype of our fluorescence imaging-based CD4 cell counter, Helios CD4 Analyzer (Helios). We also conducted blood tests with the Helios and compared the results with the data obtained using the PIMA.

## 2. Technical Development

### 2.1. Fabrication of Sample Cartridge

**Figure 1:**
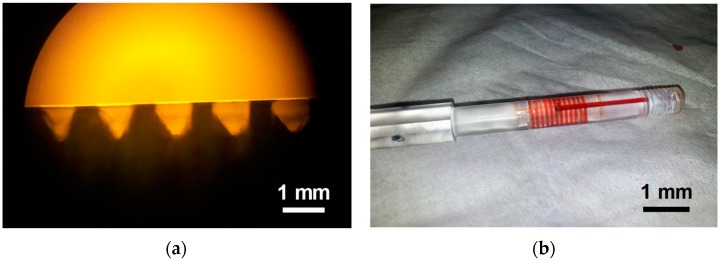
(**a**) Cross-section of a helical minichannel formed by a conventional screw thread and transparent adhesive tape. (**b**) Custom-made sample cartridge filled with whole blood.

As illustrated in Figure 1a, a conventional screw thread covered by a transparent adhesive tape (Scotch^®^ Transparent Tape Dispenser Roll, 18 mm × 3 m, 3M, St. Paul, MN, USA) can form a helical minichannel with a variable channel width and depth. We invented a sample cartridge with a thread-like microgroove formed on the cylindrical surface. Figure 1b depicts a custom-made sample cartridge filled with whole blood. Blood was introduced in the helical minichannel through a hole at the end of the screw. As the channel has a pre-defined width and depth, the detection volume is determined solely by the number of sample images acquired for the analysis [8]. The concentration of cells can be estimated by dividing the cell count with the sample volume.

**Figure 2:**
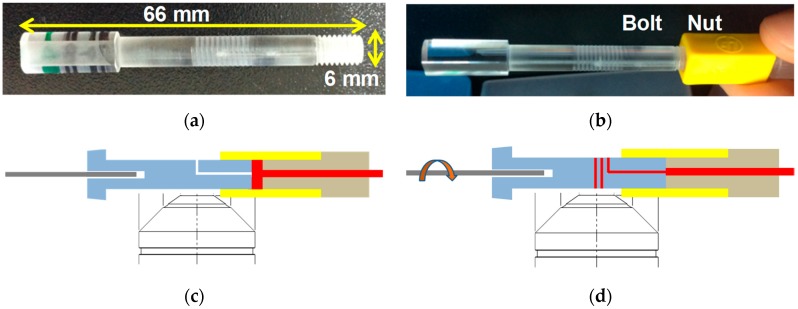
(**a**) Photo of a bolt with a helical minichannel. (**b**) Photo of the bolt connected to a nut. (**c**,**d**) Working principle of nut and bolt microfluidics. Finger-pricked whole blood (red) fills the space inside the nut (yellow). By rotating the bolt (blue) in the clockwise direction, the blood is introduced to the helical minichannel through a hole made at the end of the bolt.

Figure 2a,b illustrate the fabricated sample cartridge with the helical minichannel. The cartridge has a length of 66 mm and diameter of 6 mm. The height and width of the helical minichannel are 100 μm and 600 μm, respectively. Figure 2c,d depict the working principle of nut-bolt microfluidics. The foot area of the sample cartridge with the 3D helical minichannel is smaller than that of the conventional sample cartridge with a planar microchannel.

### 2.2. Fluorescence Imaging Setup

**Figure 3:**
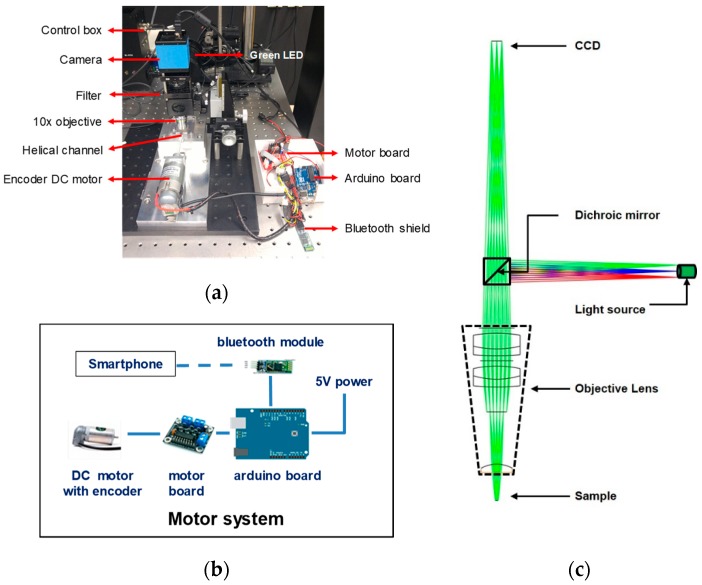
(**a**) Experimental setup equipped with a DC motor and Arduino board to control the nut and bolt microfluidic chamber. The control box is connected to a light source. (**b**) Motor system comprising an Arduino board and a DC motor controlled by a smartphone wirelessly. (**c**) Schematic of the fluorescence imaging setup for ray tracing simulation. Wavelength of light source is in the range of 530–550 nm. Excitation and emission filters are placed in front of the excitation light source and the CCD sensor, respectively.

The nut and bolt microfluidic imaging system is equipped with a light-emitting diode (LED) module which has an emission wavelength at 530 nm, a CCD (charge-coupled device) camera, filters for excitation and emission (595 nm for CD4 and 695 nm for CD3 detection) as illustrated in Figure 3a. For spinning, the helical minichannel was fixed to a DC motor with along an encoder for improving the control of rotation velocity. An Arduino board with a Bluetooth shield controlled the DC motor so that the rotation velocity and time could be controlled over a Bluetooth connection using a smartphone, as shown in Figure 3b.

Fluorescently-labeled particles or cells were excited using a green-colored LED light focused with a 10×/0.3 NA objective lens, while the emitted fluorescence signals through the filter set were detected using the CCD camera with a resolution of 1280 × 960 pixels. The camera was synchronized with the motor as well as LED using an external pulse/delay signal generator, and it acquired multiple images one by one whenever the motor was rotated by 20 degrees.

All the components used to build the fluorescence imaging setup have been listed in Table 1. As the image is taken from the convex surface of the cylindrical sample cartridge, optical distortion may be caused by the field curvature effect. To resolve this issue, an optic simulation with ray tracing was performed for the schematic layout depicted in Figure 3c; the result will be described in Section 4. With a compact fluorescence imaging setup, the implemented nut-bolt mechanism allows scanning of relatively large volume of the sample along the helical channel by just rotating the cylindrical chamber using a coupled single DC motor in stepping mode, which greatly simplifies related electromechanical parts.

### 2.3. Optimization of Fluorescence Imaging Parameters

The quantification of fluorescence signals has become an important tool for analyzing cellular structures and functions [12]. However, a precise measurement or count of the fluorescence signal from an image mostly depends on the combination of optical imaging system components. A systematic optimization procedure was carried out for finding the best optical component assembly.

#### 2.3.1. Objective Lens

**Figure 4:**
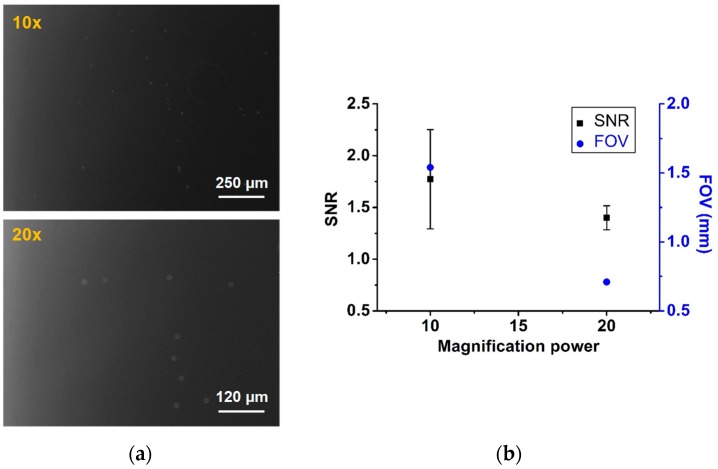
(**a**) Fluorescence images taken with 10× and 20× objectives, respectively. (**b**) SNR and FOV measurement for different lenses while the distance was kept fixed between the camera and the sample chamber.

A compact fluorescence microscope was built in our lab facility to check the performance of signal detection sensitivity based on the resolution and sensitivity of the camera. A series of quantitative analyses was performed to find the best combination of optical parts. To capture a low light signal emitted from the fluorescently-labeled CD4 cell, high-sensitivity camera is advantageous; hence, we used several sensitive cameras to test their performance. For the quantitative study, we used a camera with 0.05 lux (lx) sensitivity but changed the objective lens to understand its effect on the image as illustrated in Figure 4. Two commercially available objective lenses (10× and 20× magnification) were used and a higher signal-to-noise ratio (SNR) and bigger field of view (FOV) were obtained with the 10× lens. The SNR is measured by the signal gray value (intensity) over background gray value (intensity) of the raw image as given below.

*SNR* = fluorescence signal intensity/background intensity (1)

#### 2.3.2. Field-of-view

**Figure 5:**
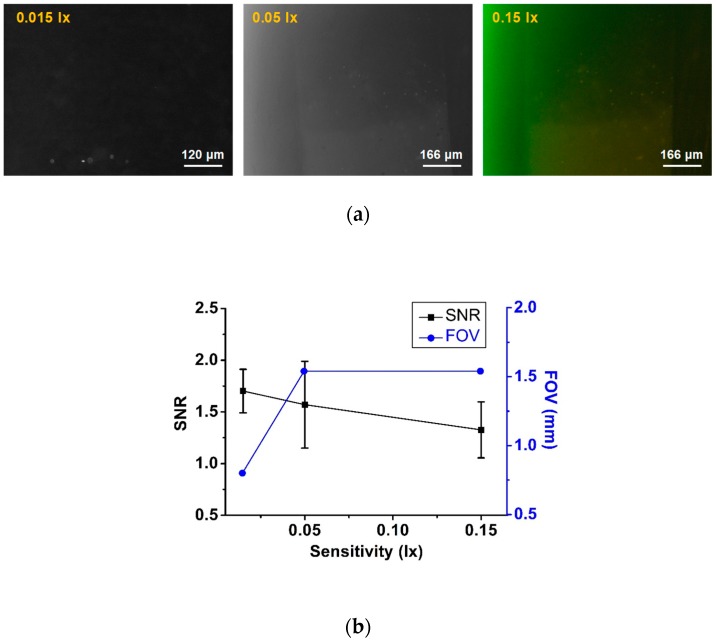
(**a**) Fluorescence images taken using high- and low-sensitivity cameras. (**b**) Low-resolution and high-sensitivity camera exhibits a higher SNR compared to a high-resolution and low-sensitivity camera.

We used three cameras to demonstrate the sensitivity effect in our study. In the images captured using a highly sensitive and low-resolution camera with 0.015 lx (DMK21BU618, Imaging Source, Bremen, Germany), we found that the SNR was high in a small FOV. However, the other two cameras having sensitivities of 0.05 lx (DMK51BU02, Imaging Source, Bremen, Germany) and 0.15 lx (DFK51BU02, Imaging Source, Bremen, Germany), respectively, showed the same FOV as they had the same resolution; meanwhile, the low-sensitivity camera showed less SNR, as is illustrated in Figure 5a. Figure 5b plots the SNR measurement with respect to the camera specification.

The higher SNR measured using the higher-sensitivity camera could be considered as a trivial finding in general. However, the exposure time (0.1 s) was kept as low as possible in our study to detect the weakly fluorescent CD4 cells. Increasing exposure time would lead to higher background and lower SNR, accordingly, as the signal is very low in the CD4 images. In contrast, an image of beads with strong fluorescence still exhibits higher SNR even in this low exposure condition. Our intension was to keep the background as dark as possible to avoid fluorescence signal saturation.

#### 2.3.3. Light Power

**Figure 6:**
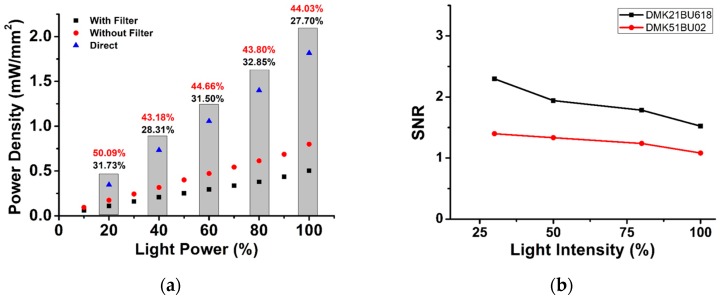
(**a**) Power density measured at different light powers. (**b**) SNR for different light intensities.

It should be noted that the excitation light power at the sample influences the detection level. SNR is one of the important factors to be considered for detecting fluorescent particles. Here we have optimized various imaging parameters to find the best condition for ensuring high SNR. SNR is calculated using the ratio between the fluorescence signal intensity and background of the image intensity, as given in Equation (1). Higher SNR indicates better contrast of the signal from the background. It appears that increasing the light power given to the sample increases the signal visibility. In conventional microscopy, light passes through several filters before reaching the image sensor. In this process, it loses some power as illustrated in Figure 6a. Approximately half of the light power was lost during the travel from the source to the camera sensor in our measurement. A lower light power at the sample gives us high SNR, as shown in Figure 6b.

We switched on the light only when we captured the image. As we did not keep the light source on for a long time, the photobleaching effect was minimized. By increasing the light power, SNR decreases because the background intensity increased.

### 2.4. Prototype Development

**Figure 7:**
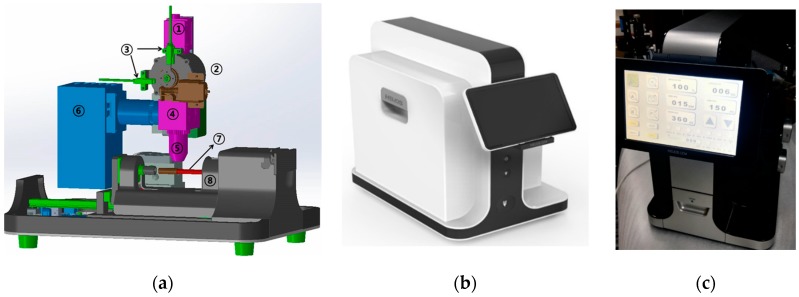
(**a**) Schematic of the optical module in the prototype. 1. CCD camera, 2. Fluorescence filter container, 3. Sensors for filter rotation, 4. Dichroic mirror, 5. 10× objective lens, 6. 530 nm LED, 7. Sample cartridge, 8. DC motor. (**b**,**c**) Complete prototype of the Helios CD4 Analyzer.

We developed a prototype following the optimization process with the experimental setup that we built using commercially available optical components. The optical module illustrated in Figure 7a comprised a CCD camera (DMK21BU618, Imaging Source, Bremen, Germany), optical filters (XF108-2 Cy3/695AF55 CY5M, Omega Optical, Brattleboro, VT, USA), an objective lens (UPLFLN10, Olympus, Tokyo, Japan), and custom-made LED. We used two emission filters to detect the fluorescence signals from CD4 and CD3 cells. These filters were inserted into a round filter container and their positions were switched. The LED was switched on using an electrical signal generated every time the DC motor rotated the sample cartridge. Thus, a pair of CD4 and CD3 images could be obtained automatically from the same FOV at the helical minichannel.

Figure 7b,c show the complete prototype. We developed an electrical control system and installed it in the prototype. A touch display connected to the electrical board was installed in the prototype so that several functions, such as motor speed control, LED light power on/off, and sample scanning could be adjusted.

## 3. Materials and Methods

### 3.1. Optical Simulation

A ray tracing simulation was performed using ZEMAX OpticsStudio 14.2 Standard version (ZEMAX LLC, Kirkland, WA, USA) with a fluorescence imaging system layout shown in Figure 3c. In the simulation, a 10× objective lens is included, and the objective consists of five single lenses. This ray tracing simulation can help analyze, model, and design an optical system to optimize the imaging performance.

### 3.2. Sample Preparation

To obtain clear sample images, blood samples were prepared by dissolving red blood cells (RBC) in RBC lysing solution (Ca. 420301, BioLegend, San Diego, CA, USA). However, the washing process was omitted to minimize the leukocyte loss. We collected fresh blood from human volunteers with permission from the institutional review board (KMU-201412-BR-043). Please note that 200 µL of the RBC lysis solution was dispensed into the sample tube, and 100 µL of blood was added to that tube. Then, the mixture was stirred slowly for 10 min at room temperature, and 10 µL of CD4 and CD3 fluorescent dyes each were injected into the sample tube. The tube was incubated in the dark for 10 min at room temperature, accompanied by slow stirring. After incubation, blood conjugated with the fluorescent dye was injected into the sample cartridge using a 1 cc syringe. The instrument was prepared for operation by inserting the sample cartridge filled with blood into the motor holder.

### 3.3. Image Processing

The concentration of particles was measured by counting the total number of particles in the pre-defined sample volume. 200 images were captured from individual samples by using two filters suitable for handling CD4 and CD3 fluorescence. These images were divided in two groups, namely CD4 images and CD3 images, and then analyzed automatically using image analysis software (ImageJ ver. 1.51r, http://imagej.nih.gov/ij/) with added plugins named “Subtract Background” and “Auto Local Threshold”. The Subtract Background plugin can correct for unevenly illuminated background by using the “Rolling Ball” algorithm, and the Auto Local Threshold plugin can binarize 8-bit images using various local thresholding methods [13]. We applied the Bernsen thresholding method in this study [14]. An alternative matrix-based approach has been proposed recently to balance non-uniform illumination in microscopy [15].

**Figure 8:**
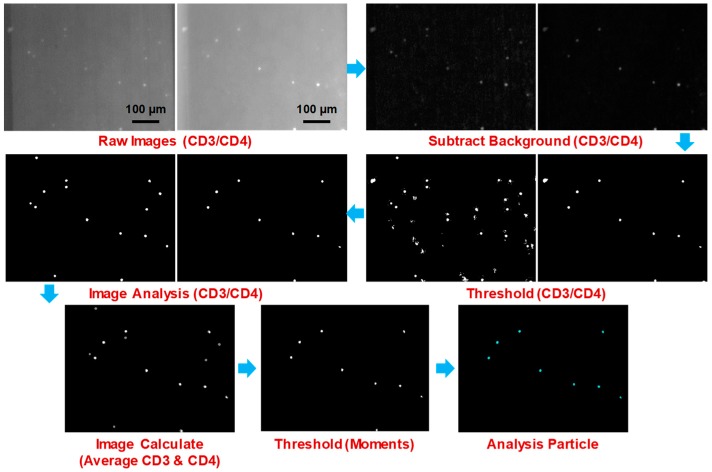
Procedure of image processing and analysis for counting CD4 cells

Every image set was analyzed and the particles in the images were counted using a set of image analysis parameters optimized for CD4 and CD3 image features. Finally, we counted the number of particles that appeared to be identical in both CD4 and CD3 images using the “Image Calculate” function in the software. A procedure for image processing and analysis for counting CD4 cells is shown in Figure 8.

The size of the image was 0.6 mm in width and 0.49 mm in height, and the channel depth was 0.1 mm. Therefore, one image contained 0.0294 µL of the blood sample. The total probe volume was linearly proportional to the number of images used for analysis. The CD4 cell count result obtained using our Helios prototype was compared to the result obtained using PIMA.

## 4. Results and Discussion

**Figure 9:**
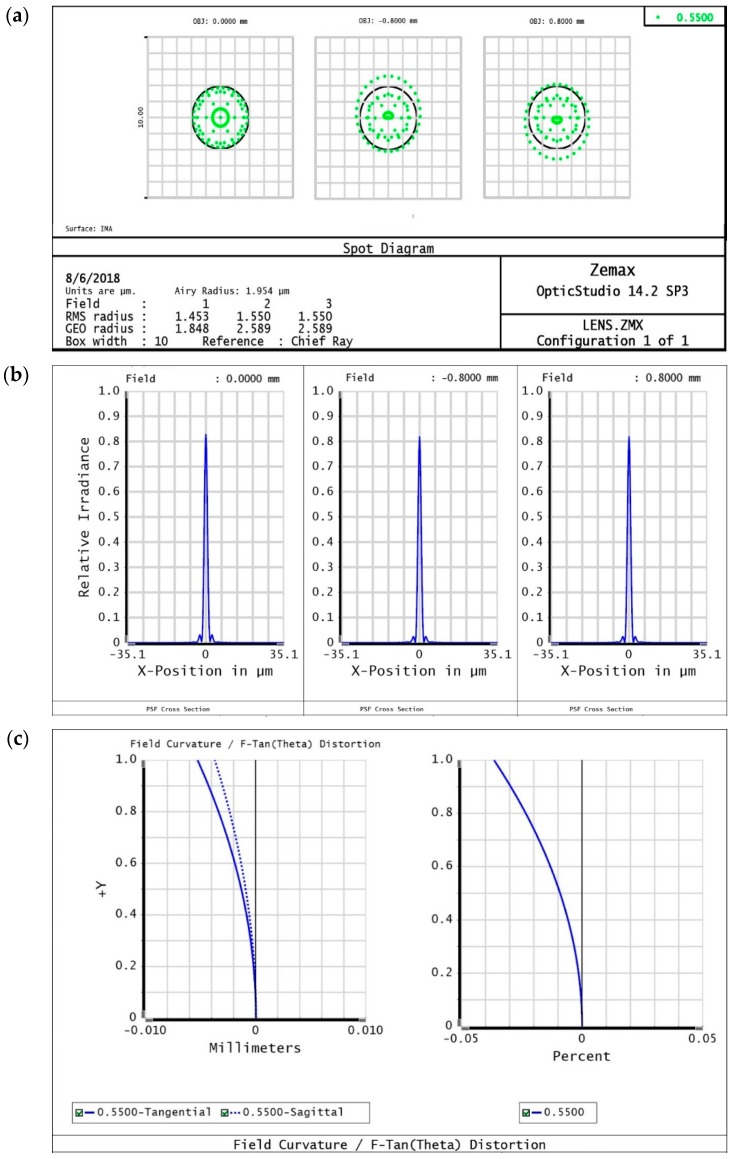
(**a**) Spot diagram with the black circle showing the Airy disk (1.95 µm) for the imaging system in the center and edge of the image field. (**b**) Cross section of the PSF shown in terms of the relative irradiance at *y*-axis. Relative intensity is approximately the same over the image field from the center to the edge. (**c**) Field curvature and percent of distortion are shown from left to right, respectively. Very small field curvature effect can be observed at the edge, which is about 4 µm, and the percent of distortion is negligible.

The spot diagram, point spread function (PSF), and field curvature and distortion show very good optical performance characteristics. The radius of the Airy disk for this system is 1.95 µm, which satisfies our theoretical measurement using the following equation:

Airy radius = 1.22 × λ × F/#(2)

where, 1.22 is diffraction limited value, λ denotes the wavelength (550 nm) of the light source, and F/# denotes f-number (2.91). The Airy disk radius is a measure to find the minimum distance between two particles that are separate from each other. This measurement shows a good resolution demonstrating that adjacent particles can be separated, as their radius is very narrow. Furthermore, the ray pattern inside the Airy disk proves that the particle signal would be circular in shape, as shown in Figure 9a.

The cross-sectional PSFs on the image plane at 550 nm are illustrated in Figure 9b. This PSF represents light intensity over the entire FOV on the imaging surface. The on-axis at the center as well as off-axis at the edge of the image plan has similar intensities. Figure 9c depicts a quantitative analysis consisting of field curvature graphs. The dark blue and dotted blue lines represent the sagittal and tangential surfaces, respectively. The on-axis position (H = 0 mm) in this graph is considered to be the center of the 1.6 mm sample surface. The focus points are deviated from the image plane with an increasing height. However, the maximum deviation at the 0.8 mm height is 4 µm, but in our imaging system the depth of focus is greater than this value. In other words, this deviation does not make any difference in the purpose of fluorescence imaging. The percentage of distortion, displayed in Figure 9c, is found to be negligible because its maximum distortion measurement is only 0.03%.

**Figure 10:**
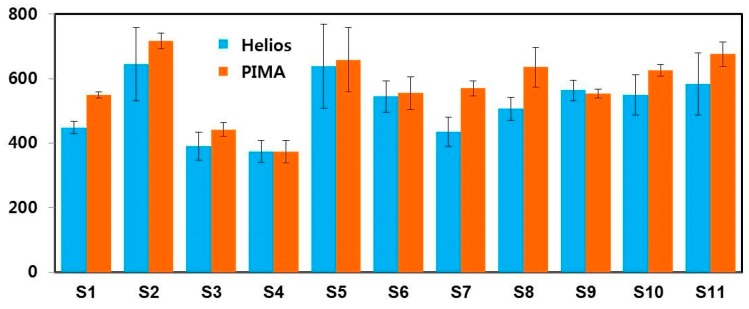
Comparison of the mean CD4 cell count obtained with Helios and PIMA.

We developed a prototype of an optical imaging-based CD4 cell analyzer and measured the CD4 count in human blood. The cell count result obtained using the Helios was compared with that of PIMA data to validate its accuracy. The reproducibility of the prototype Helios was verified by repeatedly obtaining measured data for the same sample. Figure 10 illustrates the comparison of CD4 cell counts obtained using Helios and PIMA with 11 blood samples.

There was a ±2–20% difference between the results obtained using Helios and PIMA, while the deviation in the Helios data was 1.6 times greater than that in the PIMA data in the reproducibility test. The degree of agreement was estimated using *p*-values as summarized in Table 2.

**Figure 11:**
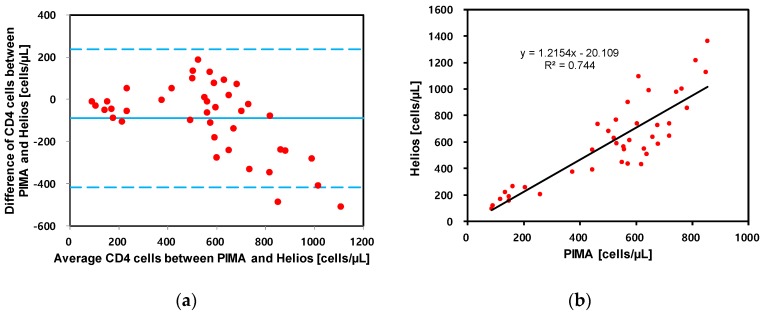
(**a**) Bland–Altman plot comparing absolute CD4 cell counts estimated by Helios and conventional PIMA. The solid blue continuous lines indicate the bias (mean difference). (**b**) Correlation plot of CD4 cell counts for Helios and PIMA.

The *p*-values ranged from 0.01 for the CD4 cell counts <200 cells/mm^3^ to 0.97 for 200–350 cells/mm^3^. The Bland–Altman plot, in Figure 11a, demonstrates that the variation in CD4 cell counts between the two methods is within the agreeable limits of ±1.96 times the standard deviation (SD). The error distribution was found to be bi-directional. The correlation of CD4 count measured using Helios and PIMA is illustrated in Figure 11b.

Helios uses one light source to detect two different fluorescence signals, and CD4 images are twice as bright as CD3 images, which might cause many particles to be lost during our image analysis. In addition, the sample volume could be less than the calculated volume if the blood sample does not completely fill the channel, which could lead to erroneous counting results. Further optimization of the image analysis parameters for analyzing CD4 and CD3 images together along with the effort to improve the image quality by stably fixing the cartridge holder in the prototype would ensure higher accuracy of the CD4 count.

The RBC lysing step in the sample preparation is time-consuming and laborious. It can also affect the sample integrity by exposing the cells to non-physiological environment [16]. Size-based continuous cell separation technique has been developed in last decade for detection of isolated pathogenic bacteria [17] and separation of leukocytes from whole blood [18] with the aid of helical or spiral microchannels. It is found that the development of Dean flow in a curved microchannel enhances the lateral migration of particles, which leads to the separation of particles [19]. Our previous computational fluid dynamics study has shown that the cross-section and pitch of the helical channel have a significant effect on Dean flow pattern [20]. Separation of white blood cells from whole blood in our helical minichannel-based sample cartridge will improve the SNR of CD4 cell images and provide a more accurate CD4 count.

We believe that the efficient management of patients can enhance the quality of human health in resource-confined regions that have many patients suffering from HIV/AIDS. This could be made possible by delivering medical services and health benefits on a larger scale using low-cost portable equipment. The total cost of a POC CD4 test depends on various individual pieces of information [6]. Our ultimate goal is to develop a helical minichannel-based compact CD4 testing module that can be integrated with a smartphone, where the phone camera, LEDs, and motor are fully controlled using an application software. Employing the proposed method, a low-cost CD4 monitoring device can be developed in the near future to replace the large and expensive analytical instruments currently being used.

## 5. Conclusions

We developed a new CD4 test platform comprising a sample cartridge with a 3D helical minichannel and fluorescence imaging module equipped with a nut and bolt scanning mechanism. Performance tests using human blood samples showed reasonable agreement with PIMA and left room for further technical improvement in terms of the CD4 count. The WHO currently recommends the ART initiation if the CD4 counts are less than 350 cells/μL. Our Helios CD4 analyzer would enable CD4 test results to be rapidly obtained using a drop of blood in a POC setting, which can greatly decrease the time delay from diagnosis to initiation of the ART and thus reduce mortality from HIV/AIDS.

## Figures and Tables

**Table 1:** Components used in the fluorescence imaging setup.

Component	Model No.	Company
Arduino Board	Arduino Uno (R3)	LK EMBEDDED, Seoul, Korea
Bluetooth Shield	HC-06	LK EMBEDDED, Seoul, Korea
Camera	DMK21BU618	Imaging Source, Bremen, Germany
DC Encoder Motor	MB-3352E-0521-24V	Robotmart, Seoul, Korea
Filters	XF108-2 Cy3/695AF55 CY5M	Omega Optical, Brattleboro, VT, USA
Light-Emitting Diode	Custom-made Green LED with Controller	BoardLab, Incheon, Korea
Motor board	AM-DC1-3D	NEWTC, Seoul, Korea
Motor Power Supply	PS-2425	Robotmart, Seoul, Korea
Objective lens	UPLFLN10 × 2	Olympus, Tokyo, Japan

**Table 2:** *p*-Values and limit of agreement for absolute CD4 cell counts determined using Helios.

CD4+ T-Cell Group	Bland–Altman Difference between PIMA and Helios (Limits of Agreement)		T-Test (*p*-Value)
<200	−48.15 (−114.76–18.46)	*n* = 7	0.01
200–350	−1.76 (−105.94–102.43)	*n* = 2	0.97
350–500	−80.28 (−325.55–162.99)	*n* = 4	0.34
>500	−109.03 (−485.57–267.51)	*n* = 27	0.00
Total	−90.14 (−416–236.34)	*n* = 40	0.00
